# Drug use evaluation: A two‐year retrospective review of the effectiveness and tolerability of agomelatine versus mirtazapine in patients with depressive disorder

**DOI:** 10.1002/brb3.2311

**Published:** 2021-08-01

**Authors:** Shek Ming Leung

**Affiliations:** ^1^ Institute of Vocational Education ‐ Kwai Chung Campus New Territory Hong Kong

**Keywords:** agomelatine, depressive disorder, mirtazapine, retrospective studies

## Abstract

**Objective:**

To compare the effectiveness and tolerability of agomelatine with mirtazapine in patients with depressive disorder. To illustrate the prescribing pattern of agomelatine and identify factors that affect the pattern of treatment result and therapeutic outcome of it.

**Methods:**

The clinical data of patients using agomelatine or mirtazapine, 93 patients in each group, were included and reviewed in this retrospective study. Background characteristics, adverse events, therapeutic outcomes (discontinued or continued), reason of discontinuation, and the presence of positive pattern of treatment result were assessed. Positive pattern of treatment result was defined as either recovery or improvement of depressive disorder after therapy.

**Results:**

Patients using agomelatine were associated with higher starting dose and higher dose titrated than mirtazapine. More patients started agomelatine due to intolerability, and less due to ineffectiveness of the previous antidepressant. More patients started agomelatine before the use of at least two selective serotonin reuptake inhibitor (SSRI)/serotonin‐noradrenaline reuptake inhibitor (SNRI). Patients using agomelatine were associated with less discontinuation due to intolerability, and less experience of adverse events within 90 days of initiation or dose increase, but more discontinuation due to ineffectiveness versus mirtazapine. The use of 50 mg resulted in less discontinuation. The use of at least two SSRI(s)/SNRI(s) before and more concomitant medications are independently associated with more discontinuation due to intolerability. The use of at least two SSRI(s)/SNRI(s) before was also associated with more adverse events. Using agomelatine as an augmentation to other antidepressant(s) and at a higher dose were independently associated with the experience of positive pattern of treatment result.

**Conclusion:**

Agomelatine was more tolerable than mirtazapine, but could result in more discontinuation due to ineffectiveness. The use of higher dose and as an augmentation to other antidepressant(s) could improve the desired treatment result of agomelatine.

## Graphical abstract

This retrospective study compared the effectiveness and tolerability of agomelatine with mirtazapine in patients with depressive disorder, and identified the factors affecting treatment outcome of agomelatine. Agomelatine was found to be more tolerable than mirtazapine, but had more discontinuation due to ineffectiveness. Increasing the dose to 50 mg or using it as an augmentation to other antidepressant(s) were associated with more positive outcome.

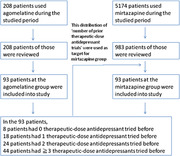

## 1 INTRODUCTION

Agomelatine is an antidepressant with novel mechanism of action, and is also classified as an atypical antidepressant. It is an agonist at melatonin receptors (MT_1_ and MT_2_) that could restore circadian rhythm and improve sleeping quality (Green, 2011; Hickie & Rogers, 2011; Kasper et al., 2010). It is also a 5HT_2C_ receptor antagonist that increases the release of dopamine and noradrenaline at frontal cortex (Green, 2011; Hickie & Rogers, 2011; Kasper et al., 2010). Without affecting the extracellular level of serotonin and affinity of other receptors, it lacks discontinuation symptoms and many side effects of traditional antidepressants like selective serotonin reuptake inhibitors (SSRIs) and serotonin‐noradrenaline reuptake inhibitors (SNRIs) (Green, 2011; Hickie & Rogers, 2011; Kasper et al., 2010).

Agomelatine can also improve the sleeping quality in depressed patients. Studies showed that agomelatine could significantly increase sleep efficiency, reduce the sleep latency, and improve subjective sleeping quality; at the same time, improve daytime functioning, circadian rest–activity cycle, and reduce daytime sleepiness, when being compared with SSRIs and SNRIs (Kasper et al., 2010; Lemoine et al., 2007; Quera‐Salva et al., 2011). In terms of sleep architecture, unlike tricyclic antidepressants and SSRIs, agomelatine did not affect rapid eye movement (REM) sleep (Quera‐Salva et al., 2011), which was suggested to cause physiological abnormality and affect memory and learning if inhibited. A study also revealed that agomelatine was also a well‐tolerated alternative to treat generalized anxiety disorder (GAD) (Stein et al., 2008).

With the above properties, agomelatine had become an antidepressant that attracted researchers. Nevertheless, there were obstacles for researchers to evaluate it. Most of the head‐to‐head comparison trials were using SSRIs or SNRIs as comparators, but there were few, if any, studies comparing agomelatine with other atypical antidepressants (Guaiana et al., 2013). The clinical role of agomelatine among atypical antidepressants was not clear.

Mirtazapine is another atypical antidepressant with a similar role as agomelatine. It is a noradrenergic and specific serotonergic antidepressant, that increases central noradrenaline and serotonin by central presynaptic alpha‐2 antagonism; it also possesses antagonism on 5HT_2_, 5HT‐_3_, and H_1_ receptors (Schering Corporation, 2009). It was also recommended as a third‐line antidepressant, like agomelatine, after the failure of SSRIs and SNRIs in The Maudsley Guidelines (Taylor et al., 2015). It also shared other similarities with agomelatine in its clinical role, like improving sleep without affecting the REM sleep (Aslan et al., 2002; Winokur et al., 2000), treating GAD (Falkai, 1999; Schatzberg, 2000), and lacking side effect of sexual impairment (Serretti & Chiesa, 2009). Therefore, mirtazapine was a good candidate for comparison with agomelatine.

The primary objective of this study was to compare the effectiveness/desired treatment result and tolerability of agomelatine with mirtazapine, in patients with depressive disorder, through a retrospective review of 2‐year electronic clinical data in Castle Peak Hospital, a psychiatric hospital under Hospital Authority in Hong Kong. The secondary objective was to illustrate the pattern of use in real‐world setting and identify any factors affecting the pattern of treatment result and therapeutic outcome of agomelatine.

## 2 METHODS

### 2.1 Study design, procedure, and sample recruitment

A retrospective study was conducted by reviewing the electronic patient records of patients prescribed with agomelatine from June 1, 2016 to May 31, 2018, drawn by the Clinical Data Analysis and Reporting System at Castle Peak Hospital. Ethical approval was obtained from Hospital Authority (Reference no.: NTWC/REC/19009) and the University of Sunderland (application no.: 003108). For the control group, the same way was used to recruit patients prescribed with mirtazapine in the same period. Each patient was assigned with a random number as an identifier.

Patients were eligible if they had at least one recorded psychiatric follow‐up after prescribing agomelatine before the end of studied period; and had a diagnosis of depressive disorder mentioned in the electronic patient record by doctor, defined by the Diagnostic and Statistical Manual of Mental Disorder, 5^th^ edition (DSM‐5), which includes disruptive mood dysregulation disorder, major depressive disorder, persistent depressive disorder (dysthymia), and substance/medication‐induced depressive disorder (American Psychiatric Association, 2013), at the time of starting agomelatine (or mirtazapine) recorded in the electronic patient record.

Patients who used agomelatine before the start of studied period were excluded. Those without a diagnosis of depressive disorder recorded at the time of starting agomelatine were also excluded. Patients were also excluded if they had a diagnosis of bipolar affective disorder. If the patients were not prescribed with a therapeutic dose, at least 25 mg/day, of agomelatine, they were also excluded. Patients prescribed with as‐needed dose of agomelatine were also excluded. Patients were also excluded if agomelatine was not initiated at Hospital Authority. Patients who did not take agomelatine after it had been prescribed, reported in the electronic patient record, were also excluded.

Patients with other concurrent mental disorder(s), except bipolar affective disorder, were not excluded, in order to reflect the real‐world practice, as co‐morbidity of mental disorders was common in reality.

The above inclusion and exclusion criteria were also applied on the mirtazapine group, with its therapeutic dose defined as at least 15 mg/day.

All patients in the agomelatine group fulfilling the inclusion and exclusion criteria were recruited into study. After that, the number of antidepressants tried before agomelatine was reviewed. Antidepressants used at subtherapeutic dose were not counted as a trial of antidepressant (Table 1). If the patient had a 1‐year antidepressant‐free period, the antidepressants used before that period were not counted. Patients were then categorized as four levels: “no antidepressant tried before,” “one antidepressant tried before,” “two antidepressants tried before,” and “three or more antidepressants tried before.”

**TABLE 1:** Subtherapeutic doses of antidepressants

Antidepressants	Subtherapeutic doses (mg/day)	Subtherapeutic doses in elderly (i.e., >65 years old) (mg/day)
Trazodone	<150	<100
Nortriptyline	<75	<30
Amitriptyline	<75	<50
Trimipramine	<75	<50
Dothiepin	<75	<50
Clomipramine	<75	<30
Imipramine	<75	<30
Mirtazapine	<15	<15
Mianserin	<30	<30
Citalopram	<20	<10
Escitalopram	<10	<5
Fluoxetine	<20	<20
Sertraline	<50	<50
Paroxetine	<20	<20
Fluvoxamine	<50	<50
Milnacipran	<100	<100
Desvenlafaxine	<50	<50
Venlafaxine	<75	<75
Duloxetine	<60	<60
Vortioxetine	<10	<5
Bupropion	<150	<150
Agomelatine	<25	<25

(Allergan, 2017a, 2017b; Eli Lilly & Company, 2010, 2017; Emc, 2018a, 2018b, 2020a, 2020b, 2021; Excellium Pharmaceutical, Inc., 2012; GlaxoSmithKline, 2009, 2012; Jazz Pharmaceuticals, 2008; Mallinckrodt Inc., 2007a, 2007b; Mylan, 2013, 2019; NHS, 2018; Pfizer, 2011, 2016, 2017; Pragma Pharmaceuticals, LLC., 2017; Sandoz, 2014; Schering Corporation, 2009; Servier, 2018; Spencer & Wilde, 1998; Takeda Pharmaceuticals America, Inc., 2014; Taylor et al., 2015; Teva Select Brands, 2014; Tignol et al., 1998; Valentine, 1976).

Patients list of the mirtazapine group were sorted according to the identifiers and randomized by picking identifiers at regular interval of 10. The distributions of prior therapeutic‐dose antidepressant trials for both arms were matched by excluding the cases in mirtazapine group once the number of patients for that level was achieved.

### 2.2 Data collection

Background data were recorded, which included the reason of starting agomelatine (or mirtazapine), either as “Intolerability to the previous antidepressant,” “Ineffectiveness of the previous antidepressant,” or “Other reason(s)” (to be specified and recorded according to individual cases), number of concomitant regularly used systematic medications, gender, age of the patient when agomelatine (or mirtazapine) was prescribed, duration of therapy within the study period, starting dose of agomelatine (or mirtazapine), highest dose of agomelatine (or mirtazapine) tried before the end of study period, whether the patient used at least two SSRI(s)/SNRI(s) at any dose before agomelatine (or mirtazapine), and whether agomelatine (or mirtazapine) was started as an antidepressant monotherapy or as an augmentation to other antidepressant(s).

It was also recorded whether agomelatine (or mirtazapine) was discontinued or continued before the end of the study period. For those who discontinued it, the reason was recorded, either as “Intolerability to the medication,” “Ineffectiveness of the medication” (including the lack of response or insufficient response), “Noncompliance to the medication,” “Resolution of depression,” or “Other reason(s)” (to be specified and recorded according to individual cases). For those who continued it, the patterns of treatment result were recorded. The patients were assigned as either “Recovered,” “Improved” (including improvement in any of the depressive symptoms noted in DSM‐5), “Depressive symptoms maintained” (including the lack of response or insufficient response), or “Not mentioned.” The electronic patient records of all the follow‐up after the initiation of treatment were reviewed until the end of study period, May 31, 2018. The categorization of the above was based on how the doctor mentioned it in the electronic patient record. The patient was categorized as “Recovered” if the doctor mentioned any related term, such as “recovery” and “remission” in the electronic patient record. If the doctor mentioned any improvement in any depressive symptom, such as improvement in insomnia, the patient was categorized as “Improved.” If the doctor mentioned that the patient was still having similar depressive symptoms without mentioning any desired change of the depressive symptoms, the patient was categorized as “Depressive symptoms maintained.” If the doctor did not mention anything about the depressive symptoms in the record, the patient was categorized as “Not mentioned.” If there were more than one follow‐up after the initiation of treatment, an overall pattern of treatment was summarized. For example, if the depressive symptoms were initially mentioned to be improved, followed by worsening of symptoms mentioned in later follow‐up, the overall pattern of treatment was summarized by comparing the depressive symptoms mentioned in the latest record with those mentioned in the initial record. Whether an augmentation (“starting of” or “dose increase of” another concomitant antidepressant, antipsychotic with antidepressant effect, or lithium) was needed before the end of the study period was also recorded for those continuing therapy. The adverse effects experienced within 90 days of starting therapy or dose increase were also recorded. If the patient discontinued the therapy, the adverse events causing discontinuation were also recorded.

Effectiveness/desired treatment result was measured as the following three parameters: 1) the number of patients with positive pattern of treatment result, which included “Improved” and “Recovered” in those continued therapy and “Resolution of depression” in those discontinued therapy; 2) the number of patients who discontinued therapy due to “Ineffectiveness”; and 3) the number of patients continued therapy but needed an augmentation to it.

Tolerability wasmeasured as the following two parameters: 1) the number of patients discontinued therapy due to “Intolerability”; and 2) the number of patients experiencing adverse effects within 90 days of starting therapy or dose increase.

### 2.3 Statistical analysis

Descriptive statistics were presented as percentage for discrete variables and means +/− standard deviation (SD) for continuous variables. Independent sample *t*‐test and chi‐square test were performed for patients’ characteristics. Logistic regression models, adjusting for age, gender, number of concomitant regularly used systematic medications, number of previous antidepressant trials, percentage of British National Formulary (BNF) maximum for the starting dose, percentage of BNF maximum for the highest dose tried within the study period, whether the medication was started as antidepressant monotherapy or augmentation to other antidepressant(s), and duration of therapy, was used to compare the three effectiveness/desired treatment result parameters and the two tolerability parameters of agomelatine with mirtazapine. To identify factors affecting the pattern of treatment result and therapeutic outcomes of agomelatine, logistic regression models were employed with background data as independent variables. Odd ratio (OR) and 95% confidence interval (CI) were calculated with the logistic regression models. A *p*‐value of less than .05 was considered significant. All statistical analyses were performed using SPSS version 22.0 (Armonk, NY: IBM Corp.).

The primary outcome, comparison of effectiveness/desired treatment result and tolerability between agomelatine and mirtazapine, was measured by using logistic regression, adjusted for baseline characteristics, on the three parameters of effectiveness/desired treatment result and the two parameters of tolerability mentioned above. The secondary outcome, identifying any factors affecting the pattern of treatment result and therapeutic outcome of agomelatine, was measured by using logistic regression, with baseline characteristics employed.

## 3 RESULTS

### 3.1 Patients’ background characteristics and pattern of use

**FIGURE 1:**
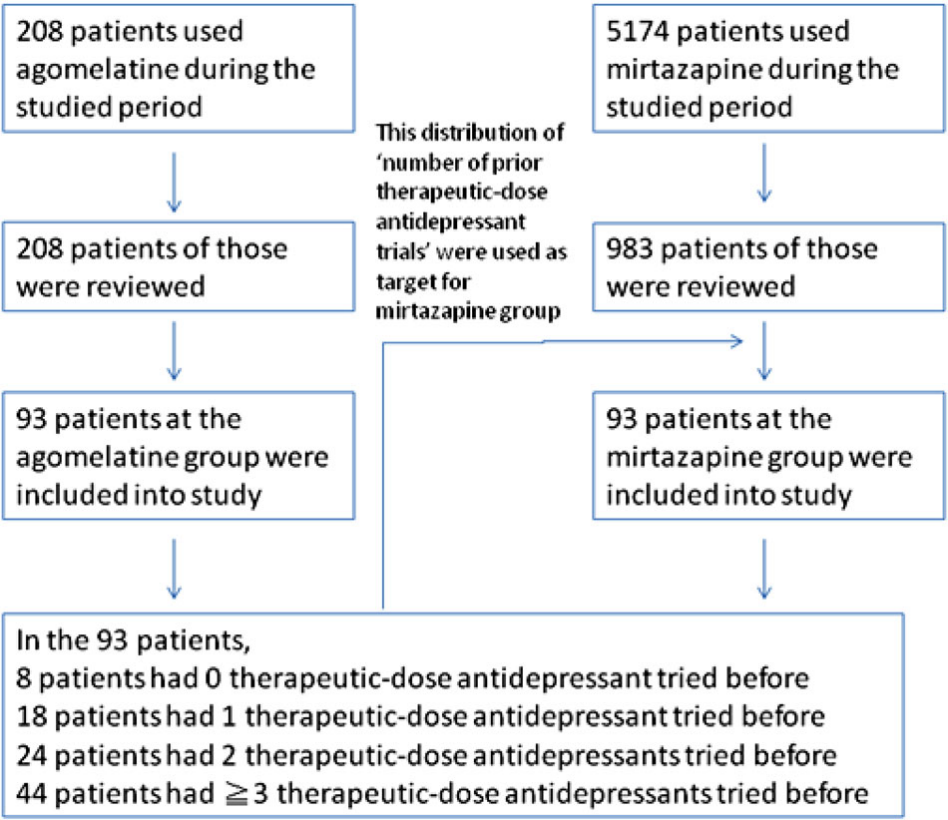
Selection of studied patients

During the 2‐year study period, a total of 208 patients used agomelatine. All of their clinical data within the studied period were reviewed, with 93 (44.7%) of the 208 patients included into the study for analysis. Ninety‐three among 5174 patients at the mirtazapine group that were included into the study with the distribution of prior therapeutic‐dose antidepressant trials matched (Figure 1).

Table 2 shows the patients’ background characteristics for patients using agomelatine and mirtazapine. None of the patients in both groups was diagnosed with substance/medication‐induced depressive disorder. Patients using agomelatine had a significantly shorter duration of therapy (152 ± 144 days vs. 268 ± 217 days, *p* < .001). Patients using agomelatine had a relatively higher starting dose (27.7 ± 7.8 mg; 55.4 ± 15.6% vs. 14.6 ± 3.2 mg; 32.4 ± 7.1%, *p* < .001) and highest dose tried (36.3 ± 12.5 mg; 72.6 ± 25.0% vs. 24.9 ± 11.4 mg; 55.4 ± 25.4%, *p* < .001) in terms of percentage of BNF maximum. In the agomelatine group, more patients started the therapy due to intolerability to the previous antidepressant (33.3% vs. 17.2%, *p* < .05), and less patients started the therapy due to ineffectiveness of the previous antidepressant (53.8% vs. 73.1%, *p* < .01). Less patients in the agomelatine group tried at least two SSRI(s)/SNRI(s) before the therapy (54.8% vs. 69.9%, *p* < .05) when compared to patients using mirtazapine. The mean number of therapeutic‐dose antidepressant trials for the agomelatine group was 2.8 ± 2.0, with no significant difference from the patients using mirtazapine. No difference in the gender, age, number of concomitant regularly used systemic medications, the use of therapy as antidepressant monotherapy were found between patients using agomelatine and mirtazapine.

**TABLE 2:** Patients’ background characteristics

	Agomelatine (*n* = 93)	Mirtazapine (*n* = 93)	*p*‐value
Male, *n* (%)	26 (28)	29 (31)	.63
Mean age ± SD (years)	47.6 ± 12.9	51.0 ± 13.6	.08
Mean duration of therapy ± SD (days)	152 ± 144	268 ± 217	<.001
Mean number of prior therapeutic‐dose antidepressant trials ± SD (*n)*	2.8 ± 2.0	2.3 ± 1.3	.09
Mean number of concomitant regularly used systemic medications ± SD (*n)*	2.8 ± 2.8	2.4 ± 2.5	.29
Starting as monotherapy, *n* (%)	54 (58.1)	49 (52.7)	.46
Mean starting dose ± SD (mg)	27.7 ± 7.8	14.6 ± 3.2	
Mean percentage of BNF maximum of starting dose ± SD (%)	55.4 ± 15.6	32.4 ± 7.1	<.001
Starting dose, *n* (%)			
*Agomelatine*, 25 mg	83 (89.2)		
*Agomelatine*, 50 mg	10 (10.8)		
*Mirtazapine*, 7.5 mg		9 (9.7)	
*Mirtazapine*, 15 mg		82 (88.2)	
*Mirtazapine*, 30 mg		2 (2.2)	
Mean highest dose tried ± SD (mg)	36.3 ± 12.5	24.9 ± 11.4	
Mean percentage of BNF maximum of highest dose tried ± SD (%)	72.6 ± 25.0	55.4 ± 25.4	<.001
Highest dose tried, *n* (%)			
*Agomelatine*, 25 mg	51 (54.8)		
*Agomelatine*, 50 mg	42 (45.2)		
*Mirtazapine*, 15 mg		48 (51.6)	
*Mirtazapine*, 30 mg		28 (30.1)	
*Mirtazapine*, 37.5 mg		1 (1.1)	
*Mirtazapine*, 45 mg		16 (17.2)	
Reason of starting therapy			
Intolerability to the previous antidepressant, *n* (%)	31 (33.3)	16 (17.2)	<.05
Ineffectiveness of the previous antidepressant, *n* (%)	50 (53.8)	68 (73.1)	<.01
Other reason(s), *n* (%)	12 (12.9)	9 (9.7)	.49
Trial of at least two SSRI(s)/SNRI(s) before, *n* (%)	51 (54.8)	65 (69.9)	<.05

Abbreviations: SNRI(s), serotonin‐noradrenaline reuptake inhibitor(s); SSRI(s), selective serotonin reuptake inhibitor(s).

Some patients had a duration of treatment shorter than 2 weeks. In the agomelatine group, four patients discontinued the treatment in 2 weeks; in the mirtazapine group, three patients discontinued the treatment in 2 weeks.

Among 12.9% patients starting agomelatine for reasons other than intolerability and ineffectiveness of the previous therapy, 3.2% started it due to noncompliance to the previous antidepressant; 2.2% started agomelatine as the first antidepressant for co‐morbid insomnia; and 2.2% started it due to co‐morbidity of glaucoma (type not mentioned). No specific reason was mentioned in the electronic patient records for the other 5.4% patients.

### 3.2 Pattern of treatment result and therapeutic outcome of agomelatine

Table 3 shows the pattern of treatment result and therapeutic outcome of agomelatine, compared with mirtazapine, after adjusting for background characteristics. Patients using agomelatine were significantly less likely to discontinue therapy due to intolerability (OR, 0.19; 95%CI, 0.06–0.58; *p* < .01) and more likely to discontinue therapy due to ineffectiveness (OR, 5.26; 95%CI, 1.12–24.79; *p* < .05) when compared with mirtazapine. Nevertheless, there was no significant difference in the overall discontinuation rate among the two groups, with 39.8% of patients using agomelatine discontinued therapy within the studied period. There were significantly less patients using agomelatine who experienced adverse events within 90 days of initiation or dose increase (OR, 0.30; 95%CI, 0.10–0.89; *p* < .05) versus mirtazapine. There was no significant difference in terms of rate of positive pattern of treatment result and the need of augmentation therapy among the two groups.

**TABLE 3:** Pattern of treatment result and therapeutic outcome of patients using agomelatine and mirtazapine

	Agomelatine (*n* = 93)	Mirtazapine (*n* = 93)	OR^a^	95% CI
Continued therapy, *n* (%)	56 (60.2)	58 (62.4)	1.44	0.55–3.82
Continued therapy, recovered, *n* (%)	0	0	NA^c^	NA^c^
Continued therapy, improved, *n* (%)	29 (31.2)	34 (36.6)	0.87	0.34–2.2
Continued therapy, depressive symptoms maintained, *n* (%)	23 (24.7)	23 (24.7)	1.37	0.50–3.78
Continued therapy, pattern of treatment not mentioned, *n* (%)	4 (4.3)	1 (1.1)	12.85	0.19–859.26
Discontinued therapy, *n* (%)	37 (39.8)	35 (37.6)	0.69	0.26–1.83
Discontinued therapy, intolerability, *n* (%)	15 (16.1)	25 (26.9)	0.19	0.06–0.58**
Discontinued therapy, ineffectiveness, *n* (%)	17 (18.3)	4 (4.3)	5.26	1.12–24.79*
Discontinued therapy, noncompliance, *n* (%)	3 (3.2)	1 (1.1)	763.82	NA^c^
Discontinued therapy, resolution of depression, *n* (%)	0	2 (2.2)	2878.47	NA^c^
Discontinued therapy, other reason(s), *n* (%)	2 (2.2)	3 (3.2)	180.17	NA^c^
With adverse events within 90 days of initiation or dose increase, *n* (%)	21 (22.6)	27 (29.0)	0.30	0.10–0.89*
With positive pattern of treatment result ^b^, *n* (%)	29 (31.1)	36 (38.7)	0.82	0.32–2.09
Needing augmentation when continued, *n* (%)	8 (8.6)	17 (18.3)	0.57	0.13–2.56

Abbreviations: CI, confidence interval; NA, not applicable: OR, odds ratio.

^*^ *p* < .05 versus mirtazapine.

^**^ *p* < .01 versus mirtazapine.

^a^Odds ratio was calculated with logistic regression adjusted for gender, age, duration of therapy, number of prior therapeutic‐dose antidepressant trials, number of concomitant regularly used systemic medications, percentage of BNF maximum of starting dose, percentage of BNF maximum of highest dose tried and whether therapy was started as an antidepressant monotherapy.

^b^ Positive pattern of treatment result included “improved” and “recovered” in those continued therapy, and “resolution of depression” in those discontinued therapy.

^c^ Not applicable due to too few occurrences.

Two (2.2%) patients discontinued agomelatine due to other reasons. One of them attributed diarrhea and tremor to agomelatine, which were not resolved after discontinuation and therefore were not regarded as adverse events by the prescriber. The prescriber of the other patient discontinued agomelatine when alanine transaminase was found to be mildly elevated, with its level lower than three times of upper limit of normal. Nevertheless, since the alanine transaminase level was the same as baseline, it was not regarded as an adverse event from agomelatine.

### 3.3 Adverse events profile for agomelatine

Table 4 shows the adverse events causing discontinuation of therapy in patients using agomelatine. The most common adverse events leading to discontinuation were dizziness and gastrointestinal side effects (nausea, vomiting, or stomach discomfort), both with frequencies of 3.2%. Table 5 shows the adverse events experienced within 90 days of initiation or dose increase of agomelatine. The most common adverse event was dizziness (6.5%). Tables 6 and 7 show the adverse events leading to discontinuation and adverse events within 90 days of initiation or dose increase for mirtazapine. Patients using agomelatine had a lower frequency of oversedation (2.2% vs. 12.9%), weakness, tiredness or fatigue (3.2% vs. 6.5%), and weight gain (0 vs. 3.2%) than patients using mirtazapine within 90 days of initiation or dose increase.

**TABLE 4:** Adverse events causing discontinuation of therapy in patients using agomelatine

Adverse events	Frequencies
Adverse events within 90 days of initiation or dose increase	14 (15.1%)
Dizziness	3 (3.2%)
Nausea, vomiting, or stomach discomfort	3 (3.2%)
Headache	2 (2.2%)
Blurred vision	2 (2.2%)
Oversedation	2 (2.2%)
Abdominal discomfort	2 (2.2%)
Poor sleep	1 (1.1%)
Nightmare	1 (1.1%)
Hangover	1 (1.1%)
Weakness	1 (1.1%)
Chest discomfort	1 (1.1%)
Mood fluctuation	1 (1.1%)
Anxiety	1 (1.1%)
Rash	1 (1.1%)
Adverse events beyond 90 days of initiation or dose increase	1 (1.1%)
Mildly elevated alanine transaminase (<3 × upper limit of normal, with normal level at baseline)	1 (1.1%)

**TABLE 5:** Adverse events experienced within 90 days of initiation or dose increase of agomelatine

Adverse events	Frequencies
Dizziness	6 (6.5%)
Nausea, vomiting or stomach discomfort	3 (3.2%)
Weakness or tiredness	3 (3.2%)
Poor sleep	3 (3.2%)
Blurred vision	3 (3.2%)
Headache	2 (2.2%)
Oversedation	2 (2.2%)
Abdominal discomfort	2 (2.2%)
Chest discomfort	1 (1.1%)
Mood fluctuation	1 (1.1%)
Too stimulated	1 (1.1%)
Anxiety	1 (1.1%)
Hangover	1 (1.1%)
Pain (parotid region)	1 (1.1%)
Constipation	1 (1.1%)
Nightmare	1 (1.1%)
Rash	1 (1.1%)
Increased suicidal ideation	1 (1.1%)
Palpitation	1 (1.1%)

**TABLE 6:** Adverse events causing discontinuation of therapy in patients using mirtazapine

Adverse events	Frequencies
Adverse events within 90 days of initiation or dose increase	22 (23.7%)
Oversedation	11 (11.8%)
Weakness, tiredness, or fatigue	5 (5.4%)
Weight gain	3 (3.2%)
Increased appetite	2 (2.2%)
Dizziness	2 (2.2%)
Palpitation	2 (2.2%)
Hand tremor	2 (2.2%)
Ankle swelling	1 (1.1%)
Restlessness	1 (1.1%)
Rash	1 (1.1%)
Generalized discomfort	1 (1.1%)
Hangover	1 (1.1%)
Headache	1 (1.1%)
Dry mouth	1 (1.1%)
Nightmare	1 (1.1%)
Constipation	1 (1.1%)
Chest discomfort	1 (1.1%)
Adverse events beyond 90 days of initiation or dose increase	3 (3.2%)
Headache	1 (1.1%)
Weight gain	1 (1.1%)
Oversedation	1 (1.1%)

**TABLE 7:** Adverse events experienced within 90 days of initiation or dose increase of mirtazapine

Adverse events	Frequencies
Oversedation	12 (12.9%)
Weakness, tiredness, or fatigue	6 (6.5%)
Weight gain	3 (3.2%)
Hangover	3 (3.2%)
Increased appetite	2 (2.2%)
Dizziness	2 (2.2%)
Palpitation	2 (2.2%)
Dry mouth	2 (2.2%)
Hand tremor	2 (2.2%)
Restlessness	1 (1.1%)
Rash	1 (1.1%)
Stomach discomfort	1 (1.1%)
Generalized discomfort	1 (1.1%)
Dry nose	1 (1.1%)
Headache	1 (1.1%)
Nightmare	1 (1.1%)
Constipation	1 (1.1%)
Ankle swelling	1 (1.1%)
Chest discomfort	1 (1.1%)

### 3.4 Factors affecting pattern of treatment result and therapeutic outcome of agomelatine

Table 8 shows that discontinuation of the use of agomelatine was independently associated with 25 mg when compared with 50 mg (OR, 0.13; 95%CI, 0.04–0.45; *p* < .01) after adjusting for other factors, including gender, age, duration, number of prior antidepressant trials, whether the patient tried two SSRI(s)/SNRI(s) before, number of concomitant medications, starting dose, reason of starting, and whether it was started as monotherapy or augmentation. Although shorter duration of therapy also had statistically significant association with higher discontinuation rate, the effect was not clinically significant as the OR was close to 1 (OR, 1.00; 95%CI, 0.99–1.00; *p* < .05).

**TABLE 8:** The association of discontinuation with patients’ background characteristics in patients using agomelatine

	Discontinued therapy			
	Yes (*n* = 37)	No (*n* = 56)	OR	95% CI
Gender, *n* (%)				
Male	9 (24.3)	17 (30.4)	0.61	0.18–2.07
Female	28 (75.7)	39 (69.6)	1.0	
Mean age ± SD (years)	47.6 ± 14.0	47.6 ± 12.3	0.97	0.93–1.02
Mean duration of therapy (days) ± SD	101 ± 140	186 ± 137	1.00	0.99–1.00*
Mean number of prior therapeutic‐dose antidepressant trials ± SD (*n)*	2.9 ± 1.8	2.6 ± 2.2	0.99	0.71–1.38
Trial of at least two SSRI(s)/SNRI(s) before, *n* (%)				
Yes	22 (59.5)	29 (51.8)	1.39	0.37–5.24
No	15 (40.5)	27 (48.2)	1.0	
Mean number of concomitant regularly used systemic medications ± SD (*n)*	3.2 ± 2.8	2.6 ± 2.9	1.22	0.99–1.51
Starting dose, *n* (%)				
25 mg	34 (91.9)	49 (87.5)	1.0	
50 mg	3 (8.1)	7 (12.5)	1.87	0.27–12.87
Highest dose tried, *n* (%)				
25 mg	29 (78.4)	22 (39.3)	1.0	
50 mg	8 (21.6)	34 (60.7)	0.13	0.04–0.45**
Reason of starting therapy, *n* (%)				
Intolerability to the previous antidepressant	12 (32.4)	19 (33.9)	1.49	0.26–8.56
Ineffectiveness of the previous antidepressant	22 (59.5)	28 (50.0)	3.42	0.63–18.59
Starting as, *n* (%)				
Antidepressant monotherapy	21 (56.8)	33 (58.9)	2.60	0.77–8.82
Augmentation to other antidepressant(s)	16 (43.2)	23 (41.1)	1.0	

Abbreviations: CI, confidence interval; OR, odds ratio; SD, standard deviation; SNRI(s), serotonin‐noradrenaline reuptake inhibitor(s); SSRI(s), selective serotonin reuptake inhibitor(s).

* *p* < .05 versus mirtazapine.

** *p* < .01 versus mirtazapine.

In terms of tolerability, in patients using agomelatine, Table 9 shows that discontinuation due to intolerability was independently associated with the trial of at least two SSRI(s)/SNRI(s) before (OR, 11.98; 95%CI, 1.21–118.40; *p* < .05) and more concomitant regularly used systemic medications (OR, 1.50; 95%CI, 1.03–2.19; *p* < .05) after adjusting for other background characteristics. Table 10 shows that, adverse events within 90 days of initiation or dose increase of agomelatine was also independently associated with the trial of at least two SSRI(s)/SNRI(s) before (OR, 8.12; 95%CI, 1.29–51.3; *p* < .05). Again, although shorter duration of therapy was also statistically associated with adverse events within 90 days of initiation or dose increase, its effect was not clinically significant as OR was too close to 1 (OR, 0.99; 95%CI, 0.98–1.00; *p* < .05).

**TABLE 9:** The association of discontinuation due to intolerability with patients’ background characteristics in patients using agomelatine

	Discontinuation due to intolerability			
	Yes (*n* = 15)	No (*n* = 78)	OR	95% CI
Gender, *n* (%)				
Male	5 (33.3)	21 (26.9)	1.29	0.27–6.28
Female	10 (66.7)	57 (73.1)	1.0	
Mean age ± SD (years)	48.3 ± 12.9	47.4 ± 13.0	1.00	0.93–1.07
Mean duration of therapy (days) ± SD	96 ± 182	163 ± 134	1.00	0.99–1.00
Mean number of prior therapeutic‐dose antidepressant trials ± SD (*n)*	2.7 ± 1.2	2.8 ± 2.2	0.58	0.30–1.10
Trial of at least two SSRI(s)/SNRI(s) before, *n* (%)				
Yes	11 (73.3)	40 (51.3)	11.98	1.21–118.40*
No	4 (26.7)	38 (48.7)	1.0	
Mean number of concomitant regularly used systemic medications ± SD (*n)*	3.7 ± 3.2	2.7 ± 2.8	1.50	1.03–2.19*
Starting dose, *n* (%)				
25 mg	13 (86.7)	70 (89.7)	NA^a^	NA^a^
50 mg	2 (13.3)	8 (10.3)	1.0	
Highest dose tried, *n* (%)				
25 mg	13 (86.7)	38 (48.7)	NA^a^	NA^a^
50 mg	2 (13.3)	40 (51.3)	1.0	
Reason of starting therapy, *n* (%)				
Intolerability to the previous antidepressant	6 (40.0)	25 (32.1)	NA^a^	NA^a^
Ineffectiveness of the previous antidepressant	9 (60.0)	41 (52.6)	NA^a^	NA^a^
Starting as, *n* (%)				
Antidepressant monotherapy	8 (53.3)	46 (59.0)	2.92	0.43–19.7
Augmentation to other antidepressant(s)	7 (46.7)	32 (41.0)	1.0	

Abbreviations: CI, confidence interval; NA, not applicable; OR, odds ratio; SD, standard deviation; SNRI(s), serotonin‐noradrenaline reuptake inhibitor(s); SSRI(s), selective serotonin reuptake inhibitor(s).

* *p* < .05 versus mirtazapine.

^a^ Not applicable due to too few occurrences.

**TABLE 10:** The association of adverse events within 90 days of initiation or dose increase with patients’ background characteristics in patients using agomelatine

	Adverse events within 90 days of initiation or dose increase			
	Yes (*n* = 21)	No (*n* = 72)	OR	95% CI
Gender, *n* (%)				
Male	6 (28.6)	20 (27.8)	0.93	0.22–3.98
Female	15 (71.4)	52 (72.2)	1.0	
Mean age ± SD (years)	46.9 ±12.1	47.8 ± 13.2	0.97	0.92–1.03
Mean duration of therapy (days) ± SD	65 ± 89	177 ± 147	0.99	0.98–1.00*
Mean number of prior therapeutic‐dose antidepressant trials ± SD (*n)*	2.5 ± 1.7	2.8 ± 2.1	0.63	0.40–1.00
Trial of at least two SSRI(s)/SNRI(s) before, *n* (%)				
Yes	14 (66.7)	37 (51.4)	8.12	1.29–51.3*
No	7 (33.3)	35 (48.6)	1.0	
Mean number of concomitant regularly used systemic medications ± SD (*n)*	3.1 ± 3.3	2.8 ± 2.7	1.21	0.94–1.57
Starting dose, *n* (%)				
25 mg	19 (90.5)	64 (88.9)	1.0	
50 mg	2 (9.5)	8 (11.1)	1.89	0.14–26.31
Highest dose tried, *n* (%)				
25 mg	17 (81.0)	34 (47.2)	1.0	
50 mg	4 (19.0)	38 (52.8)	0.18	0.03–1.27
Reason of starting therapy, *n* (%)				
Intolerability to the previous antidepressant	7 (33.3)	24 (33.3)	0.25	0.03–1.90
Ineffectiveness of the previous antidepressant	10 (47.6)	40 (55.6)	0.26	0.36–1.91
Starting as, *n* (%)				
Antidepressant monotherapy	12 (57.1)	42 (58.3)	1.71	.37–7.96
Augmentation to other antidepressant(s)	9 (42.9)	30 (41.7)	1.0	

Abbreviations: CI, confidence interval; OR, odds ratio; SD, standard deviation; SNRI(s), serotonin‐noradrenaline reuptake inhibitor(s); SSRI(s), selective serotonin reuptake inhibitor(s).

* *p* < .05 versus mirtazapine.

In terms of effectiveness and desired treatment result, in patients using agomelatine, Table 11 shows that higher chance of positive pattern of treatment result was independently associated with the use of 50 mg (OR, 7.26; 95%CI, 2.24–23.5; *p* < .001) when compared with 25 mg, and the use of agomelatine as an augmentation to other antidepressant(s) when compared with the use of it as antidepressant monotherapy (OR, 0.26; 95%CI, 0.07–0.93; *p* < .05). None of the factors was found to be associated with discontinuation due to ineffectiveness, as shown in Table 12.

**TABLE 11:** The association of positive pattern of treatment result with patients’ background characteristics in patients using agomelatine

	Positive pattern of treatment result			
	Yes (*n* = 29)	No (*n* = 64)	OR	95% CI
Gender, *n* (%)				
Male	8 (27.6)	18 (28.1)	1.52	0.44–5.18
Female	21 (72.4)	46 (71.9)	1.0	
Mean age ± SD (years)	50.0 ± 12.7	46.5 ± 13.0	1.05	0.99–1.10
Mean duration of therapy (days) ± SD	170 ± 127	144 ± 151	1.00	1.00–1.00
Mean number of prior therapeutic‐dose antidepressant trials ± SD (*n)*	2.3 ± 1.8	3.0 ± 2.1	0.98	0.66–1.45
Trial of at least two SSRI(s)/SNRI(s) before, *n* (%)				
Yes	12 (41.4)	39 (60.9)	0.26	0.06–1.17
No	17 (58.6)	25 (39.1)	1.0	
Mean number of concomitant regularly used systemic medications ± SD (*n)*	3.0 ± 2.7	2.8 ± 2.9	0.91	0.73–1.14
Starting dose, *n* (%)				
25 mg	25 (86.2)	58 (90.6)	1.0	
50 mg	4 (13.8)	6 (9.4)	1.09	0.19–6.36
Highest dose tried, *n* (%)				
25 mg	9 (31.0)	42 (65.6)	1.0	
50 mg	20 (69.0)	22 (34.4)	7.26	2.24–23.5***
Reason of starting therapy, *n* (%)				
Intolerability to the previous antidepressant	9 (31.0)	22 (34.4)	1.05	0.18–6.02
Ineffectiveness of the previous antidepressant	16 (55.2)	34 (53.1)	0.98	0.18–5.32
Starting as, *n* (%)				
Antidepressant monotherapy	15 (51.7)	39 (60.9)	0.26	0.07–0.93*
Augmentation to other antidepressant(s)	14 (48.3)	25 (39.1)	1.0	

Abbreviations: CI, confidence interval; OR, odds ratio; SD, standard deviation; SNRI(s), serotonin‐noradrenaline reuptake inhibitor(s); SSRI(s), selective serotonin reuptake inhibitor(s).

* *p* < .05 versus mirtazapine.

*** *p* < .001 versus mirtazapine.

**TABLE 12:** The association of discontinuation due to ineffectiveness with patients’ background characteristics in patients using agomelatine

	Discontinuation due to ineffectiveness			
	Yes (*n* = 17)	No (*n* = 76)	OR	95% CI
Gender, *n* (%)				
Male	3 (17.6)	23 (30.3)	0.47	0.10–2.32
Female	14 (82.4)	53 (69.7)	1.0	
Mean age ± SD (years)	48.2 ± 16.0	47.5 ± 12.3	0.99	0.93–1.04
Mean duration of therapy (days) ± SD	84 ± 85	167 ± 150	0.99	0.99–1.00
Mean number of prior therapeutic‐dose antidepressant trials ± SD (*n)*	3.1 ± 2.3	2.7 ± 2.0	1.27	0.89–1.83
Trial of at least two SSRI(s)/SNRI(s) before, *n* (%)				
Yes	8 (47.1)	43 (56.6)	0.34	0.07–1.67
No	9 (52.9)	33 (43.4)	1.0	
Mean number of concomitant regularly used systemic medications ± SD (*n)*	3.0 ± 2.6	2.8 ± 2.9	1.04	0.83–1.30
Starting dose, *n* (%)				
25 mg	16 (94.1)	67 (88.2)	1.0	
50 mg	1 (5.9)	9 (11.8)	0.44	0.04–5.61
Highest dose tried, *n* (%)				
25 mg	11 (64.7)	40 (52.6)	1.0	
50 mg	6 (35.3)	36 (47.4)	1.13	0.27–4.64
Reason of starting therapy, *n* (%)				
Intolerability to the previous antidepressant	6 (35.3)	25 (32.9)	0.98	0.14–7.06
Ineffectiveness of the previous antidepressant	9 (52.9)	41 (53.9)	1.63	0.24–10.98
Starting as, *n* (%)				
Antidepressant monotherapy	11 (64.7)	43 (56.6)	2.01	0.52–7.76
Augmentation to other antidepressant(s)	6 (35.3)	33 (43.4)	1.0	

Abbreviations: CI, confidence interval; OR, odds ratio; SD, standard deviation; SNRI(s), serotonin‐noradrenaline reuptake inhibitor(s); SSRI(s), selective serotonin reuptake inhibitor(s).

## 4 DISCUSSION

This retrospective study revealed the pattern of use of agomelatine in real practice. It showed that, compared with mirtazapine, agomelatine was more likely to be started and titrated to a relatively higher dose. The practice of prescribing a starting dose larger than the recommendation of manufacturer occurred more often for agomelatine than mirtazapine. Also, agomelatine was more often to be titrated to the maximum dose than mirtazapine. This showed that prescribers tended to be prescribing agomelatine more aggressively than prescribing mirtazapine. One of the reasons for this practice might be that, agomelatine was thought to be more tolerable than mirtazapine, which was true and was also shown in this study. This study also found that agomelatine was more likely to be started before the trial of at least two SSRI(s)/SNRI(s) as recommended by the Maudsley Guidelines, versus mirtazapine. More patients were prescribed with agomelatine due to intolerability than ineffectiveness of the previous antidepressant. These patterns were significantly different from that of patients prescribed with mirtazapine. These findings suggest that prescribers tended to start agomelatine earlier, probably due to its favorable side effect profile, especially when the patients were sensitive to side effects from another antidepressant. In short, agomelatine prescribing tended to be more aggressive and earlier, and was thought to be the treatment of choice when side effect was a concern, showing that the favorable side effect profile did affect the prescribing practice of prescribers.

From the other literatures, agomelatine was shown to have a favorable side effect profile when compared with traditional antidepressants. It did not cause gastrointestinal side effects, weight gain, cardiovascular toxicity, and sexual side effects (Kennedy & Rizvi, 2010). Previous study showed that fewer patients discontinued treatment due to adverse events in the agomelatine group when compared with venlafaxine XR (2.2% vs 8.6%; Kennedy et al., 2008). Cochrane review also concluded that agomelatine was better tolerated than paroxetine and venlafaxine in terms of overall side effects, and showed the same level of tolerability as SSRIs (Guaiana et al., 2013). In a meta‐analysis published in 2018 comparing 21 antidepressants, agomelatine was found to be significantly more acceptable than amitriptyline, clomipramine, duloxetine, fluvoxamine, reboxetine, trazodone, and venlafaxine in head‐to‐head comparison (Cipriani et al., 2018). The meta‐analysis also compared agomelatine with mirtazapine, which showed that the acceptability (dropout rate) was similar (OR, 0.81; 95%CI 0.61–1.05). Yet, there was no direct head‐to‐head clinical trial for these two atypical antidepressants. In this study, agomelatine was associated with less discontinuation due to intolerability and less occurrence of adverse events within 90 days of initiation or dose increase, even after adjusting other background factors. These findings suggest that agomelatine was not only more tolerable than traditional antidepressants shown in previous studies, but also more tolerable than mirtazapine, with less oversedation, weakness, and weight gain, which were the main concern for mirtazapine.

The fact that agomelatine was associated with higher chance of discontinuation due to ineffectiveness versus mirtazapine, even after factors like duration of therapy and dosage were considered, might alert prescribers. Nevertheless, other parameters for effectiveness/desired treatment result, which were the rate of positive pattern of treatment result and the need for augmentation, were comparable between agomelatine and mirtazapine. From previous meta‐analysis and systematic reviews, agomelatine was shown to have comparable effectiveness when compared to other antidepressants, including mirtazapine (Cipriani et al., 2018; Guaiana et al., 2013; Taylor et al., 2014). Although most studies suggested that agomelatine was comparable to other antidepressants, one randomized, double‐blind trial showed a different result. That was a study comparing vortioxetine (*n* = 252) with agomelatine (*n* = 241) in patients with major depressive disorder with an inadequate response to a single course of SSRI or SNRI (Papakostas et al., 2018). It showed that vortioxetine was significantly superior to agomelatine in patients with previous inadequate response to a single course of SSRI, with similar tolerability. This suggested that agomelatine might not be comparably effective to all antidepressants. Yet, there was no published randomized clinical trial comparing agomelatine and mirtazapine. Clinical trial comparing these two atypical antidepressants is needed to confirm any significant difference in effectiveness.

According to some studies, the average time for onset of antidepressant action was 13 days (Katz et al., 2004; Stassen et al., 1993). In this study, although a few patients in both groups, in similar number, had the duration of treatment shorter than 2 weeks, as most of the patients in both groups completed at least 2 weeks of treatment, the assessment of clinical response was still valid.

Our present study also found that, 50 mg of agomelatine was associated with lower discontinuation rate when compared to 25 mg. The reason of this was likely due to lower antidepressant effect for 25 mg. Our study also showed that discontinuation due to intolerability was independently associated with more concomitant medications. It was reasonable that the side effects from other concomitant medications could be additive to that of agomelatine, making the patients more sensitive and intolerable. This study also revealed that discontinuation of agomelatine due to intolerability and report of adverse effects were both independently associated with the use of at least two SSRI(s)/SNRI(s) previously. The prior use of at least two SSRI(s)/SNRI(s) might indicate these patients were having resistant depression. Although the exact underlying mechanism is unclear, one of the explanations why patients with resistant depression had a lower tolerability was that, resistant patients might be more sensitive to side effects and more likely to prematurely discontinue previous antidepressants. Their side effects sensitivity might also contribute to noncompliance, diminishing the effectiveness of previous antidepressants, causing them the need of multiple antidepressant trials.

Finally, this study showed that higher dosage of agomelatine and adding it to other antidepressant(s) as augmentation were both independently associated with positive pattern of treatment result. The higher antidepressant effect from 50 mg and additive antidepressant effect from other antidepressant(s) might contribute to this finding.

The major strength of the present study was the selection of mirtazapine, a clinically similar atypical antidepressant, as the comparator; and the detailed recording of background characteristics, including number of previous antidepressants trials, dosage, reason of starting therapy, and number of concomitant medications. Nevertheless, several issues should be noted when interpreting the results of this study. First, this study was not a randomized, double‐blind controlled trial. The cause–effect relationship could not be confirmed for the different outcomes between agomelatine and mirtazapine. Second, the sample size of this study was not large enough, which might reduce the generalization of this study. Third, the clinical records depended on reporting from patients and recording by prescribers, which might have resulted in a potential reporting bias. Fourth, this study used qualitative parameters instead of quantitative parameters. Different responses were defined by prescribers, which were not standardized. This limitation was hardly eliminated for a retrospective study at Hong Kong. The large patient load at Hong Kong caused the prescribers to be highly occupied. Prescribers in Hong Kong could hardly have time to use standardized scale, like PHQ‐9, to evaluate each patient in real‐world practice. Therefore, a prospective controlled trial using standardized scale to measure the treatment result would be preferred to confirm the outcome. Moreover, the baseline characteristics of the two groups in this study had significant difference. Although the primary outcome was measured with adjustment of the baseline, a randomized controlled trial would be a more convincing way to confirm the outcome. Finally, the baseline severity of depression was not reported, which might have interaction on the clinical outcome of therapy.

## 5 CONCLUSION

Agomelatine was found to be more tolerable than mirtazapine, but more patients using agomelatine discontinued therapy due to ineffectiveness. Titrating agomelatine to 50 mg and using it as an augmentation to other antidepressant(s) were associated with more desired treatment result, while using fewer concomitant medications was associated with better tolerability.

## CONFLICT OF INTEREST

The author declares no conflict of interest. No funding was received for this study.

## TRANSPARENT PEER REVIEW

The peer review history for this article is available at https://publons.com/publon/10.1002/brb3.2311s
